# Supplementary material for: Prevalence and recurrence of pica behaviors in early childhood within the ALSPAC birth cohort
Source: Int J Eat Disord. Author manuscript; Available in PMC 2024 Mar 8. (PMC10922868; doi:10.1002/eat.24111)
Supplement: 2 [file NIHMS1957185-supplement-2.docx]

**Supplementary Table A.**

*Distribution of Parent Response to Pica Across Timepoints*

|  | **Pica Waves** | | | | |
| --- | --- | --- | --- | --- | --- |
| **Response Option** | 36 Months | 54 Months | 65 Months | 77 Months | 115 Months |
| Everyday | 23 | 5 | 16 | 11 | 7 |
| More than 1/week | 60 | 30 | 14 | 17 | 10 |
| Less than 1/week | 143 | 39 | 24 | 18 | 8 |
| Not at all | 9655 | 9423 | 8704 | 8315 | 7625 |

**Supplementary Table B.**

*Chi-Square and Fischer’s Exact Results Across Time*

|  | | Pica Waves | | | | | | | |
| --- | --- | --- | --- | --- | --- | --- | --- | --- | --- |
|  | | 36 Months  *X*^2^  (p-value) | 54 Months  *X*^2^ (p-value) | | | 65 Months  *Fischer’s Exact* (p-value) | 77 Months  *Fischer’s Exact* (p-value) | 115 Months  *Fischer’s Exact* (p-value) | |
| Sex at Birth | 0.98 (.32) | | | .05 (.83) | .15 (.70) | | 1.00 (.32) | | 6.01 (.01)** |
|  |  | | |  |  | |  | |  |
| Autism | — (.003)** | | | —  (<.001)** | —  (<.001)** | | —  (<.001)** | | —  (<.001)** |
| DD | 6.83 (.01)** | | | 16.00 (.001)** | — (.04)* | | — (<.001)** | | —  (.006)** |

*Note.* n=cases, N=total sample, CI= Confidence Interval, DD= developmental delay **= significant at .01 level, *=significant at .05 level. Cells without values beyond p-values were represented with —, and indicate Fisher’s exact test was used because cell size violations.
